# Supplementary material for: Effectiveness and Safety of Ayurvedic Medicines in Type 2 Diabetes Mellitus Management: A Systematic Review and Meta-Analysis
Source: Front Pharmacol. 2022 Jun 8;13:821810. doi: 10.3389/fphar.2022.821810 (PMC9213670; doi:10.3389/fphar.2022.821810)
Supplement: Supplementary file 1 [file DataSheet1.zip › Supplementary Material/Forest and Funnel Plots/Trigonella foenum-graecum/Insulin resistance.pdf]

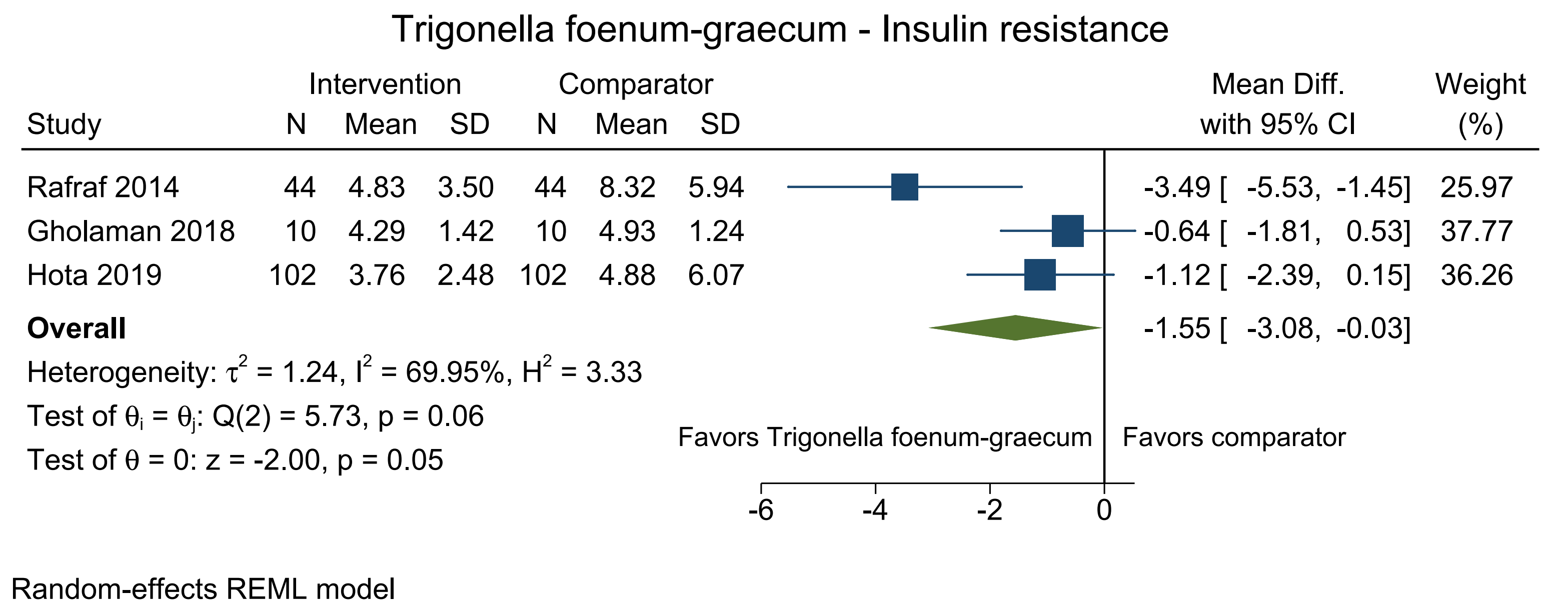
Trigonella foenum-graecum - Insulin resistance
Intervention
Comparator
Study
N
Mean
SD
N
Mean
SD
Mean Diff. with 95% CI
Weight (%)
Rafraf 2014 44 4.83 3.50 44 8.32 5.94 -3.49 [-5.53, -1.45] 25.97
Gholaman 2018 10 4.29 1.42 10 4.93 1.24 -0.64 [-1.81, 0.53] 37.77
Hota 2019 102 3.76 2.48 102 4.88 6.07 -1.12 [-2.39, 0.15] 36.26
Overall -1.55 [-3.08, -0.03]
Heterogeneity: τ^2 = 1.24, I^2 = 69.95%, H^2 = 3.33
Test of $\theta_i = \theta_j$: Q(2) = 5.73, p = 0.06
Test of θ = 0: z = -2.00, p = 0.05
Favors Trigonella foenum-graecum
Favors comparator
-6
-4
-2
0
Random-effects REML model
